# Optimisation of the Enzymatic Hydrolysis of Blood Cells with a Neutral Protease

**DOI:** 10.1155/2013/278927

**Published:** 2012-12-27

**Authors:** Yanbin Zheng, Qiushi Chen, Anshan Shan, Hao Zhang

**Affiliations:** Laboratory of Biotechnology, Institute of Animal Nutrition, Northeast Agricultural University, Harbin 150030, China

## Abstract

For utilizing the blood cells (BCs) effectively, enzymatic hydrolysis was applied to produce the enzymatically hydrolyzed blood cells (EHBCs) by using a neutral protease as a catalyst. The results of the single-factor experiments showed optimal substrate concentration, enzyme to substrate ratio (E/S), pH, temperature, and incubation period were 1.00%, 0.10, 7.00, 50.00°C, and 12.00 h, respectively. The optimized hydrolysis conditions from response surface methodology (RSM) were pH 6.50, E/S 0.11, temperature 45.00°C, and incubation period 12.00 h. Under these conditions (substrate concentration 1.00%), the degree of hydrolysis (DH) was 35.06%. The free amino acids (FAAs) content of the EHBCs (35.24%) was 40.46 times higher than BCs while the total amino acids (TAAs) content was lower than BCs. The scores of lysine (human 0.87; pig 0.97), valine (human 1.42; pig 1.38), leucine (human 1.50; pig 1.90), tyrosine (human 0.84; pig 1.09), and histidine (human 2.17; pig 2.50) indicated that the EHBCs basically fulfilled the adult human and pig nutritional requirements. The calculated protein efficiency ratios (C-PERs) of the EHBCs were 3.94, 6.19, 21.73, and 2.04. In summary, the EHBCs were produced successfully with optimized conditions and could be a novel protein source for humans and pigs.

## 1. Introduction 

 Blood is a major byproduct of abattoirs in the meat industry with large volumes [1]. It is a potentially low-cost protein source and its recycling would reduce the oxygen burden created by the biodegradation of the pollution from slaughterhouse waters [2]. The protein content of red blood cells (or blood cells) of the whole blood is 34–38% [1]. The blood cells (BCs), which are obtained by removing the plasma from whole blood, mainly contain the hemoglobin and ferrous in the form of heme-Fe with high bioavailability [3, 4]. The spray-dried blood cells are high in protein and have a favorable AA profile [5]. So, the BCs are widely used in food for animals and humans [6, 7]. However, the dark red-brown color and the metallic flavor of Hb are not desirable when it is added to some products [8]. Moreover, the BCs are difficult to be digested (due to the poor utilization of the membrane) and composed of imbalanced amino acids (high content in lysine but short of isoleucine and methionine) for animals and humans. To circumvent these problems, various attempts (e.g., enzymatic hydrolysis) have been made to shear the BC membrane and improve the functional properties of the protein isolated from the BCs [9]. Enzymatic hydrolysis could eliminate the principal color of BCs by enzymatically hydrolyzing hemoglobin [10]. The protein (nitrogen) recovery was typically 70–80% [11, 12] or more than 80% [13]. The digestibility of the enzymatically hydrolyzed proteins, which replete with FAAs and peptides of various molecular weights, is improved [13, 14], and the product is easily absorbed [15].

 The blood is frequently contaminated by contact with microorganisms and air-borne bacteria due to the application of the open systems in many industrial abattoirs but not closed collection systems [16]. So some hygienic precautions must be taken for safety during collection to prevent spoilage organisms or even pathogens from growing [16]. Moreover, the spray-drying and the high hydrostatic pressure are used to decrease the total counts of hemolyzed BC by logarithmic units [17]. The blood from animals that pass both *ante-* and *postmortem* inspections can be processed as products for animal and human [18].

The single-factor-at-a-time method is an experimental method that studies the phenomenon of interest by varying onefactorwhile fixing all other conditions. However, it is necessary to account for the influence of eachfactoron other factors and consider the interaction between thesefactors[19]. To address this problem, response surface methodology (RSM) has been proposed to determine the influences of individual factors on each other. As reported recently, statistical experimental design has been applied to optimize conditions used in many areas such as bioenergy, food, and pharmaceutical research [20–22].

The purpose of the present study was to optimize the enzymatic hydrolysis of BCs from porcine blood using a neutral protease. A central composite design (CCD; four factors and five levels) of RSM was employed to study the effects of substrate concentration, pH, temperature, E/S, and incubation period on the DH of the BCs.

## 2. Materials and Methods

### 2.1. Substrate and Enzyme

The BCs (ADDY Technology, Inc., Beijing, China) obtained from porcine blood by coagulation and drying. The neutral protease (EC 3.4.24, from *Bacillus subtilis*) purchased from Amresco is a metalloproteinase that hydrolyzes peptide bonds over a wide pH range (between 5.5 and 8.5) and exhibits maximum activity at temperatures between 45 and 50°C. 

### 2.2. Proteolytic Activity of the Enzyme

Protease activity was determined by a modification of the Casein-Pholine method [23]. One unit of protease activity was defined as the amount of enzyme that liberated 1 *μ*g of tyrosine per min at 50°C.

### 2.3. Enzymatic Hydrolysis

Prior to enzymatic treatment, a suspension of BCs was prepared by adding 9.90 mL of 0.02 mol/L buffer to 0.10 g of flour in a 20 mL glass hydrolysis tube and maintaining a constant pH value with an electrochemistry meter (Sartorius, Germany). The suspension was preheated to a constant temperature by placing the tubes in a water bath, and the enzyme was added. The enzymatic hydrolysis was conducted at a constant temperature and pH value for several hours. The tube was incubated in boiling water for 15 min to inactivate the enzyme. Then, the mixture in the tub was removed by filtration using filter paper. The filtrate was diluted to 100 mL with distilled water to determine the DH.

### 2.4. Degree of Hydrolysis

The degree of hydrolysis was expressed as the ratio of released amino nitrogen after the hydrolysis of the protein to the amount of total amino nitrogen:

(1) DH(%)=hhtot×100,

where *h* (hydrolysis equivalents) is the amount of peptide bonds cleaved during hydrolysis, which is expressed ad millimole equivalents per gram of protein (mmol/g of protein); *h*_tot_ is the total amount of peptide bonds in the protein substrate, which can be determined from the amino acid composition and is 8.62 mmol/g for BCs. 

Amino nitrogen was determined by a modified ninhydrin colorimetric method [13, 24]. A standard curve graph was generated using 2–20 *μ*g/mL hydrolyzate of the spay-dried blood cell. The procedures used to prepare hydrolyzate of the spay-dried blood cell were as follows. Fifty milligrams of spray-dried blood cells were hydrolyzed at 110°C for 24 h with hydrochloric acid (6 mol/L). The hydrolyzate was filtered and diluted to 100 mL with deionized water. One milliliter of the dilution was freeze-dried and then dissolved in deionized water at 20 *μ*g/mL. A standard curve graph was generated using 2–20 *μ*g/mL hydrolyzate. The absorbance at 570 nm was then measured against the assay components treated with deionized water as a blank. The concentration of the samples' amino nitrogen was determined according to the standard graph curve.

### 2.5. Experimental Design of RSM

Based on a single-factor experiments for DH, the proper preliminary ranges of the substrate concentration, enzyme-to-substrate ratio (E/S), pH, temperature and incubation period were determined. A five-level, four-variable CCD (Design-Expert v. 8.0.6, Stat-Ease, Inc., Minneapolis, MN, USA) was applied to determine the best combination of hydrolysis variables to maximize the DH of the BCs. Based on the single-factor experiments, the variables considered in this experimental design were the pH, temperature, E/S and incubation period. The initial conditions were pH 7.00, temperature 50.00°C, E/S 0.01, incubation period 10.00 h (the substrate concentration fixed at 1.00%).  Table 1 lists the CCD matrix and the response values that were used to develop the model. The response value in each trial was an average of triplicates.

### 2.6. Amino Acid Analysis

The free amino acid (FAA) contents were determined using an L-8900 high-speed amino acid analyzer (Hitachi, Japan) according to Kim et al. [25] with modification. Before the analysis, 5 mL of the hydrolyzates was added to 5 mL of 10% TCA. The sample was incubated at the room temperature for 2 h and centrifuged at 10000 rpm for 15 min. The supernatant collected was filtered with Millipore 0.22 *μ*m syringe filters (Milford, MA, USA). The filtrate was loaded onto the amino acid analyzer. The total amino acid (TAA) profiles of the EHBCs were determined according to AOAC [26].

The amounts of the different amino acids were expressed in mg100 mg^−1^ protein and compared with the FAO/WHO [27] and NRC [28] reference patterns. The score of essential amino acids (EAAs) was calculated as shown below:

(2) EAA  score=mg  of  EAA  in  100 mg  of  test  proteinmg  of  EAA  in  𝒜,

where *𝒜* denotes 100 mg of recommendation protein.

### 2.7. Calculated Protein Efficiency Ratio

The C-PERs were calculated using the procedures suggested by Alsmeyer et al. [29] and Lee et al. [30]. The procedures were based on the *in vitro* protein digestibility and the EAA composition of the analyzed sample. The C-PER is one of the most important scores in the evaluation of the nutritional value of proteins. It measures protein quality by feeding a diet comprising 10% of the test protein to rats and measuring their weight gain, which is an expensive and time-consuming method [31]. This method has also been applied by some researchers to predict the nutritional value of some protein hydrolyzates [21, 31, 32].

### 2.8. Statistical Analysis

 The data obtained from the CCD design were fitted with a second-order polynomial equation. The equation was as follows:

(3) Y=β0+∑i=12βiXi+∑i=12βiiXi2+∑i=1 ∑j=i+1βijXiXj,

where *Y* is the predicted response, *β*_0_ is a constant, *β*_*i*_ is the linear coefficient, *β*_*ii*_ is the quadratic coefficient, *β*_*ij*_ is the interaction coefficient, and *X*_*i*_ and *X*_*j*_ are independent variables. Data were expressed as the mean ± standard deviation (SD). The statistical significance of the model, model variables was determined at the probability (*P*) of 0.001, 0.01, or 0.05.

## 3. Results and Discussion 

### 3.1. Effects of Substrate Concentration on the DH of BCs

**Figure 1:**
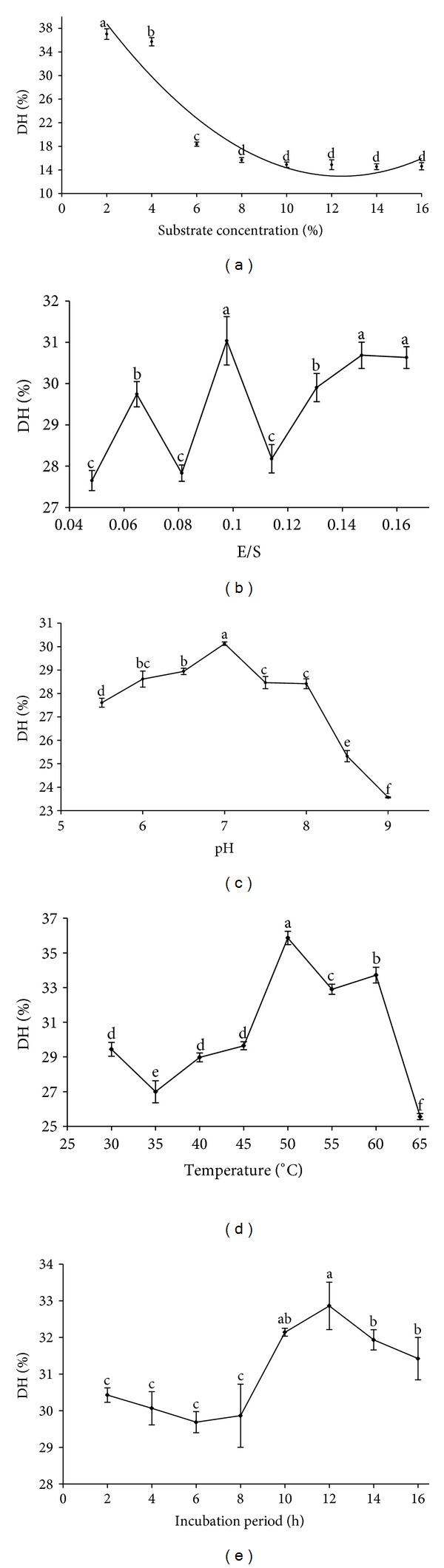
The effects of substrate concentration (a), E/S (b), pH (c), temperature (d), and incubation period (e) on the DH.

As shown in Figure 1(a) (fixed levels: E/S 0.12, pH 7.00, temperature 50.00°C, and incubation period 6.00 h), the substrate concentration (2.00%–16.00%) and the DH trended in opposite directions, that is, as the substrate concentration increased, the DH decreased according to the quadratic curve (*y* = 0.24*x* ^2^ − 5.89*x* + 49.61, *R* ^2^ = 0.90). This trend may be due to the endproduct feedback inhibition caused by hydrolysis yield [33, 34]. Taking the DH into account, the optimal substrate concentration was 1.00%, which is similar to that observed by Pérez-Gálvez et al. [9].

### 3.2. Effects of the E/S on the DH of BCs

As shown in Figure 1(b) (fixed levels: substrate concentration 1.00%, pH 7.00, temperature 50.00°C, and incubation period 6.00 h), the E/S (0.05–0.16; enzyme activity 124000 U/g) and DH varied with a similar trend. Because the cost of the enzyme contributes significantly to the total cost of the biomass conversion process [35], the E/S should be minimized as much as possible. Many reports [21, 36] noted that an increased enzyme concentration reduces the rate of hydrolysis even though the DH is increased. Considering both the cost and the DH, the optimal E/S was 0.10.

### 3.3. Effects of pH on the DH of BCs

The phosphate buffer solutions (0.02 mol/L; 5.00 < pH ≤ 8.00) and glycine-sodium hydroxide buffer solutions (0.02 mol/L; 8.00 < pH ≤ 9.00) were prepared at various pH (5.50–9.00) values. The DH by the enzyme decreased below and above the optimum pH range in a similar manner to the results observed by Hu et al. [37]. As shown in Figure 1(c) (fixed levels: substrate concentration 1.00%, E/S 0.10, temperature 50.00°C, and incubation period 6.00 h), the enzyme exhibited an optimum DH in the pH range 5.50–9.00 with the maximum DH at pH 7.00.

### 3.4. Effects of Temperature on the DH of BCs

As shown in Figure 1(d) (fixed levels: substrate concentration 1.00%, E/S 0.10, pH 7.00, and incubation period 6.00 h), the optimum hydrolysis of the BCs by the neutral protease occurred at 50.00°C. Below 50.00°C, the increased velocity of the reaction could be explained by the theory of the Arrhenius activation energy [38]. The activation energy was the key reason for the increased enzymatic reaction velocity. The DH of the protease decreased when the temperature rose above 50.00°C because of the thermal denaturation that occurs.

### 3.5. Effects of the Incubation Period on the DH of BCs

As shown in Figure 1(e) (fixed level: substrate concentration 1.00%, E/S 0.10, pH 7.00, and temperature 50.00°C), as the incubation period increases (2.00–16.00 h), the DH increased and the hydrolyzing velocity decreased. In contrast, the investigators [32, 39, 40] had reported a decrease in the DH with prolonged incubation periods. Guerard et al. [39] proposed the reduced DH observed with a prolonged incubation period might be caused by limited enzyme activity by the formation of reaction products at a high DH, the decreased concentration of peptide bonds available for hydrolysis, enzyme inhibition, and enzyme deactivation. Considering the cost, the optimal incubation period was 10.00 h. 

### 3.6. Optimisation of the Hydrolysis Parameters for DH 

#### 3.6.1. Predictive Model of Response

The influence of *X*_1_, *X*_2_, *X*_3_, and *X*_4_ on the hydrolysis by the neutral protease was determined using the factorial design described in the previous section. The best explanatory model equation for the DH value obtained from the neutral protease hydrolysis uncoded data is described in (4):

(4) DH=149.34−17.63∗  pH−2.25 ∗  Temperature+811.68∗ES−8.88 ∗  Time+0.06∗  pH  ∗  Temperature−45.74 ∗  pH∗  ES+0.26∗  pH∗  Time−  2.69 ∗Temperature∗  ES+0.03∗Temperature   ∗Time+20.88∗  ES∗Time+1.19∗pH ∗pH+0.01∗Temperature∗Temperature   −2483.73∗ES∗ES+0.22∗Time∗Time.

The experimental values and predicted values for the DH under various combinations of the independent variables are presented in Table 3. The results indicated that the DH ranged from 23.26% to 35.07%, depending on the experimental conditions (Table 2).

Statistical testing of the model was performed by ANOVA, which is required to test the significance and adequacy of the model. The *P* value for the lack of fit was not significant (*P* > 0.05), thereby confirming the validity of the model. The model was found adequate to predict within the range of the experimental variables. The coefficient values of (4) were calculated and tested for their significance using Design-Expert software and were listed in Table 3. Each *P* value is used as a tool to check the significance of each coefficient, which in turn may indicate the pattern of the interactions between the variables. It could be seen from this table that the linear coefficients (*X*_2_, *X*_3_, *X*_4_) and the quadratic term coefficient (*X*_4_) were significant with *P* values at the 0.001 level, the quadratic term coefficient (*X*_3_) was significant with a *P* value at the 0.001 level, and the cross-product coefficients (*X*_3_∗*X*_4_) were significant with a *P* value at the 0.05 level. The other term coefficients (*X*_1_, *X*_1_∗*X*_2_, *X*_1_∗*X*_3_, *X*_1_∗*X*_4_, *X*_2_∗*X*_3_, *X*_2_∗*X*_4_, *X*_1_∗*X*_1_, *X*_2_∗*X*_2_) were not significant (*P* > 0.05). The cleavage of the protein substrate's peptide bonds was markedly influenced by the hydrolysis conditions when the substrate was hydrolyzed with bacterial proteases [9, 21, 22, 38]. 

The adjusted determination coefficient (*R*_Adj_^2^) was used as the correlation measure to test the goodness of fit of the regression equation. The value of *R*_Adj_^2^ (0.90) for (4) was reasonably close to 1 and indicates a high degree of correlation between the experimental and predicted values. Good correlations of experimental results with those predicted by RSM models of proteolytic reactions had been reported by several researchers [9, 20]. The very low value of the coefficient of variation (C.V.) (3.30%) clearly indicated the very high degree of precision and reliability of the experimental values. 

**Figure 2:**
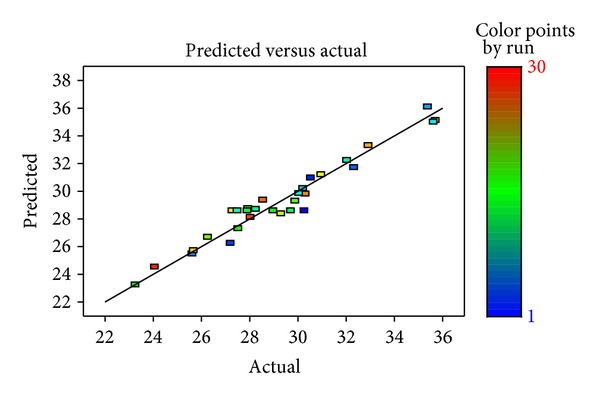
The relationship between the actual and predicted values of the DH.

Figure 2 showed the comparison between the actual values of the DH with the predicted values of the DH. The plot (Figure 2) demonstrated an acceptable level of agreement. Moreover, the coefficient (*R* ^2^ = 0.95) showed that the model was a satisfactory mathematical description of the hydrolysis process.

#### 3.6.2. Response Surface Plot and Contour Plot Showing the Effects of the Hydrolysis Variables on the DH of BCs

**Figure 3:**
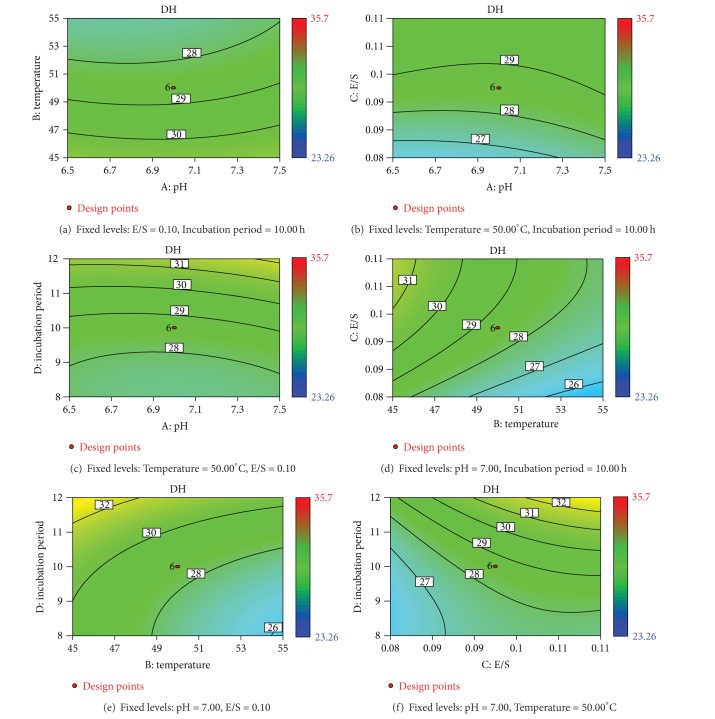
Contour plots for the effect of variables on the DH as a function of various hydrolysis conditions: (a) pH and temperature, (b) pH and E/S, (c) pH and incubation period, (d) temperature and E/S, (e) temperature and incubation period, (f) E/S and incubation period.

The graphical representations of the regression (4), known as the response surfaces and the contour plots, were presented in Figure 3. The contour plot in Figure 3(a), which presents the DH of BCs as a function of pH and temperature at fixed E/S (0.10) and incubation period (10.00 h), showed that the DH of the BCs did not vary as the pH varies from 6.50 to 7.50 and that the DH decreased as the temperature increases from 45.00 to 55.00°C. 

From Figure 3(b), it could be seen that the maximum DH of the BCs could be achieved when the pH and E/S are 6.50 and 0.11, respectively. 

From Figure 3(c), it could be seen that DH did not vary as the pH increases from 6.50 to 7.50; the DH increased steeply as the incubation period increased from 8.00 to 12.00 h. 

From Figure 3(d), it could be seen that the DH decreased as the temperature increased from 45.00 to 55.00°C; the DH increased as the E/S increased from 0.08 to 0.11. 

From Figure 3(e), it could be seen that the DH decreased as the temperature increased from 45.00 to 55.00°C, while the DH increased as the incubation period increased from 8.00 to 12.00 h. From Figure 3(f), it could be seen that the DH increased as the E/S increases from 0.08 to 0.11; the DH increased as the incubation period increased from 8.00 to 12.00 h. From Figure 3, it could be concluded that the optimal conditions for the hydrolysis of BCs with the neutral protease were pH 6.50, E/S 0.11, temperature 45.00°C, and incubation period 12.00 h. The maximum DH (35.13%) was obtained under the optimal conditions.

To ensure that the predicted result was not biased toward the practical value, experimental rechecking was performed using this deduced condition. A mean value (35.06 ± 0.06%; *n* = 3) was obtained in real experiments validated the RSM model. The good correlation between the model and these results confirmed that the response model was adequate to predict the optimisation.

### 3.7. Amino Acid Composition

The free amino acid and total amino acid composition of EHBCs (*n* = 3) and the chemical scores were presented in Table 4. The amino acids were grouped as basic (Lys, His, and Arg), acidic (Asp, Glu, and Asn), charged (basic and acidic amino acids), hydrophilic (charged amino acids, Thr, and Ser), hydrophobic (Val, Leu, Ile, Phe, Tyr, Trp, and Met), and apolar (hydrophobic amino acids except Tyr) [41]. In addition, taste attributes, as described by Tseng et al. [42], were also considered and used to categorize the amino acids as monosodium glutamate-like (MSG-like) (Asp and Glu), sweet (Ala, Gly, Ser, and Thr), bitter (Arg, His, Ile, Leu, Met, Phe, Trp, and Val) and tasteless (Cys, Lys, and Pro). After the enzymatic hydrolysis, the FAA content of the EHBCs had increased 40.46 times over that of the BCs and consisted primarily of bitter amino acids. In contrast, Guo et al. [13] reported that the high mean liberation rates of hydrophobic FAAs upon the enzymatic hydrolysis of porcine blood hemoglobin with admixture possibly decreased the bitterness. The EAA score provides an estimate of the nutritive value of a protein. This parameter compares levels of EAAs between the test and the standard proteins [32]. In the current study, the EAA scores were based on the reference protein of FAO/WHO [27] for adults and the amino acid requirements of the pig (3–10 kg), as listed by the NRC [28]. The amino acid composition in this study and its comparison with reference proteins indicated that the EHBCs levels of most of the amino acids including valine, leucine, and histidine were higher in terms of the EAAs, compared with the suggested amino acid pattern recommended by the FAO/WHO [27] for adults; some amino acids, however, were limiting in the EHBCs. Furthermore, the amino acid composition in this study and its comparison with the reference proteins indicated that the amino acids including valine, leucine, histidine, and arginine of the EHBCs were present at higher levels in terms of the EAAs, compared with the suggested amino acid pattern recommended by NRC [28] for pigs (3–10 kg). These results differed from those of Ovissipour et al. [32] because of the BC substrate's imbalanced amino acid content. The chemical score of the EHBCs showed that isoleucine and the sulfur-containingamino acids (Met and Cys) were the most limiting amino acids, while the levels of valine, leucine, phenylalanine, tyrosine, histidine and arginine exceeded the requirements for pigs (3–10 kg) [28]. Furthermore, for many animals, including pigs, diets that include large amounts of free amino acids were easily absorbed. 

The C-PER values in the current study were 2.04–21.73 for the EHBCs and lower than BCs. However, the results were higher than Yellowfin Tuna and Persian sturgeon [21, 32] due to the abundance of amino acids in the EHBCs. The C-PER values indicated the EHBC was a good potential food ingredient for adult humans and pigs (3–10 kg).

## 4. Conclusions 

The DH of BCs was studied using single-factor test and the response surface methodology to identify and quantify the variables that optimize the DH. The conditions determined by RSM for the optimal DH included the following parameters: substrate concentration 1.00%, pH 6.50, E/S 0.11, temperature 45.00°C, and incubation period 12.00 h. Basing on the EHBCs' amino acid compositions and the C-PER, the EHBCs that prepared from the BCs have a high potential for application to adult humans' food and pigs' feeds. 

## Figures and Tables

**Table 1:** The levels of the factors used to optimize the degree of hydrolysis.

Code	pH (*X*_1_)	Temperature (*X*_2_, °C)	E/S (*X*_3_)	Incubation period (*X*_4_, h)
−2	6.00	40.00	0.06	6.00
−1	6.50	45.00	0.08	8.00
0	7.00	50.00	0.10	10.00
1	7.50	55.00	0.11	12.00
2	8.00	60.00	0.13	14.00

**Table 2:** The degree of hydrolysis at various pH values, temperatures, E/S values, and incubation periods.

Run	Code values				Real values				DH*	
	*X*_1_	*X*_2_	*X*_3_	*X*_4_	*X*_1_^a^	*X*_2_^b^	*X*_3_^c^	*X*_4_^d^	Experimental	Predicted
1	0	0	0	0	7.00	50.00	0.10	10.00	30.26 ± 0.11	28.60
2	−1	−1	1	−1	6.50	45.00	0.11	8.00	30.53 ± 0.51	30.97
3	−1	1	1	−1	6.50	55.00	0.11	8.00	27.20 ± 0.38	26.25
4	−1	1	1	1	6.50	55.00	0.11	12.00	32.32 ± 0.68	31.73
5	1	1	−1	−1	7.50	55.00	0.08	8.00	25.62 ± 0.56	25.49
6	0	0	0	2	7.00	50.00	0.10	14.00	35.38 ± 0.28	36.10
7	2	0	0	0	8.00	50.00	0.10	10.00	30.21 ± 0.52	30.21
8	1	−1	1	1	7.50	45.00	0.11	12.00	35.62 ± 0.70	35.01
9	−1	−1	−1	1	6.50	45.00	0.08	12.00	30.05 ± 0.93	29.85
10	0	0	0	0	7.00	50.00	0.10	10.00	27.49 ± 0.66	28.60
11	1	1	1	1	7.50	55.00	0.11	12.00	32.03 ± 0.09	32.24
12	1	−1	−1	−1	7.50	45.00	0.08	8.00	28.25 ± 0.91	28.71
13	0	0	0	0	7.00	50.00	0.10	10.00	29.70 ± 0.34	28.60
14	0	0	0	0	7.00	50.00	0.10	10.00	27.90 ± 0.71	28.60
15	0	0	0	0	7.00	50.00	0.10	10.00	28.97 ± 0.63	28.60
16	0	0	−2	0	7.00	50.00	0.06	10.00	23.26 ± 0.95	23.25
17	−1	1	−1	1	6.50	55.00	0.08	12.00	27.52 ± 0.45	27.32
18	0	0	2	0	7.00	50.00	0.13	10.00	27.93 ± 0.67	28.77
19	1	1	−1	1	7.50	55.00	0.08	12.00	29.88 ± 0.93	29.31
20	0	2	0	0	7.00	60.00	0.10	10.00	26.26 ± 0.39	26.69
21	−1	−1	1	1	6.50	45.00	0.11	12.00	35.70 ± 0.14	35.13
22	−1	−1	−1	−1	6.50	45.00	0.08	8.00	29.30 ± 0.49	28.39
23	1	−1	−1	1	7.50	45.00	0.08	12.00	30.96 ± 0.70	31.21
24	0	0	0	0	7.00	50.00	0.10	10.00	27.28 ± 0.31	28.60
25	1	1	1	−1	7.50	55.00	0.11	8.00	25.67 ± 0.18	25.73
26	0	−2	0	0	7.00	40.00	0.10	10.00	32.92 ± 0.09	33.31
27	1	−1	1	−1	7.50	45.00	0.11	8.00	30.31 ± 0.68	29.82
28	−2	0	0	0	6.00	50.00	0.10	10.00	28.55 ± 0.39	29.38
29	0	0	0	−2	7.00	50.00	0.10	6.00	28.03 ± 0.33	28.13
30	−1	1	−1	−1	6.50	55.00	0.08	8.00	24.06 ± 0.43	24.54

*DH represents the average degree of hydrolysis of triplicate experiments. The DH was calculated using the equation *Y* = 0.69*A*_570_ − 0.01 (*R* ^2^ = 0.98) derived from the standard curve of completely hydrolyzed BCs (absorbance at 570 nm versus the concentration of the hydrolyzate).

^a^ *X*_1_: pH.

^b^ *X*_2_: temperature.

^c^ *X*_3_: E/S.

^d^ *X*_4_: incubation period.

**Table 3:** ANOVA for the response surface quadratic polynomial model.

Source	Sum of squares	df	Mean square	*F* value	*P* value	Significance
Model	0.03	14	1.88*E* − 03	20.16	<0.00	∗∗^a^
*X*_1_—pH	1.04*E* − 04	1	1.04*E* − 04	1.11	0.39	
*X*_2_—temperature	6.58*E* − 03	1	6.58*E* − 03	70.40	<0.00	∗∗
*X*_3_—E/S	4.56*E* − 03	1	4.56*E* − 03	48.84	<0.00	∗∗
*X*_4_—incubation period	9.53*E* − 03	1	9.53*E* − 03	102.04	<0.00	∗∗
*X*_1_ *X*_2_	4.00*E* − 05	1	4.00*E* − 05	0.43	0.52	
*X*_1_ *X*_3_	2.18*E* − 04	1	2.18*E* − 04	2.34	0.15	
*X*_1_ *X*_4_	1.08*E* − 04	1	1.08*E* − 04	1.15	0.30	
*X*_2_ *X*_3_	7.53*E* − 05	1	7.53*E* − 05	0.81	0.38	
*X*_2_ *X*_4_	1.74*E* − 04	1	1.74*E* − 04	1.86	0.19	
*X*_3_ *X*_4_	7.28*E* − 04	1	7.28*E* − 04	7.79	0.01	∗∗∗∗^c^
*X*_1_^2^	2.44*E* − 04	1	2.44*E* − 04	2.62	0.13	
*X*_2_^2^	3.38*E* − 04	1	3.38*E* − 04	3.62	0.08	
*X*_3_^2^	1.15*E* − 03	1	1.15*E* − 03	12.32	0.00	∗∗∗^b^
*X*_4_^2^	2.12*E* − 03	1	2.12*E* − 03	22.72	0.00	∗∗
Residual	1.40*E* − 03	15	9.34*E* − 05			
Lack of fit	6.45*E* − 04	10	6.45*E* − 05	0.43	0.88	
Pure error	7.57*E* − 04	5	1.51*E* − 04			
Cor. total	0.03	29				

Std. Dev.	9.67*E* − 03			*R*-squared	0.95	
Mean	0.29			Adj. *R*-squared	0.90	
C.V.%	3.30			Pred. *R*-squared	0.83	
PRESS	4.80*E* − 03			Adeq. precisior	18.81	

df: degree of freedom.

^a^Significance at 0.001 level.

^b^Significance at 0.01 level.

^c^Significance at 0.05 level

**Table 4:** Free amino acid and total amino acid composition of BCs and EHBCs (g/100 g) and the EAA score compared with the FAO/WHO reference protein.

	Free amino acid		Total amino acid			
Amino Acids	BCs	EHBCs	BCs	EHBCs		
Lysine	—	4.26 ± 0.05	8.16 ± 0.10	5.07 ± 0.08		
Methionine	0.19 ± 0.00	1.09 ± 0.06	0.77 ± 0.05	0.49 ± 0.02		
Threonine	0.03 ± 0.00	1.28 ± 0.06	3.00 ± 0.05	1.92 ± 0.02		
Arginine	—	3.08 ± 0.04	4.21 ± 0.11	2.79 ± 0.06		
Histidine	—	2.84 ± 0.05	7.21 ± 0.09	4.12 ± 0.06		
Isoleucine	—	—	0.19 ± 0.01	0.21 ± 0.01		
Leucine	—	6.52 ± 0.05	15.49 ± 0.20	9.90 ± 0.08		
Phenylalanine	0.06 ± 0.00	4.48 ± 0.04	6.68 ± 0.17	4.46 ± 0.06		
Valine	—	1.38 ± 0.06	7.45 ± 0.25	4.97 ± 0.05		
Tryptophan	—	—	—	—		
Serine	0.03 ± 0.00	0.56 ± 0.03	4.30 ± 0.12	2.33 ± 0.07		
Glutamic acid	0.08 ± 0.00	1.36 ± 0.04	8.06 ± 0.10	6.03 ± 0.05		
Glycine	0.33 ± 0.01	1.42 ± 0.04	4.52 ± 0.10	3.25 ± 0.05		
Proline	—	1.18 ± 0.03	3.17 ± 0.09	2.27 ± 0.07		
Cystine	0.11 ± 0.00	0.25 ± 0.01	0.31 ± 0.03	0.15 ± 0.01		
Aspartic acid	0.02 ± 0.00	1.34 ± 0.02	11.32 ± 0.54	7.81 ± 0.09		
Alanine	—	2.99 ± 0.04	7.73 ± 0.08	5.46 ± 0.06		
Tyrosine	—	1.21 ± 0.01	1.35 ± 0.05	0.85 ± 0.05		

Total	0.85 ± 0.02	35.24 ± 0.13	93.91 ± 0.06	62.08 ± 0.05		

Amino acid	EAA Score					
	Reference protein 1^a^	Reference protein 2^b^	RP1^c^		RP2^d^	
			BCs	EHBCs	BCs	EHBCs

Lysine	5.80	5.25	1.41 ± 0.02	0.87 ± 0.02	1.55 ± 0.02	0.97 ± 0.02
Threonine	3.40	3.90	0.88 ± 0.02	0.56 ± 0.01	0.77 ± 0.01	0.49 ± 0.01
Valine	3.50	3.60	2.13 ± 0.07	1.42 ± 0.01	2.07 ± 0.07	1.38 ± 0.01
Isoleucine	2.80	2.90	0.07 ± 0.00	0.08 ± 0.01	0.07 ± 0.01	0.07 ± 0.01
Leucine	6.60	5.20	2.35 ± 0.03	1.50 ± 0.01	2.98 ± 0.04	1.90 ± 0.02
Phenylalanine and tyrosine	6.30	4.85	1.27 ± 0.04	0.84 ± 0.00	1.66 ± 0.05	1.09 ± 0.01
Tryptophan	1.10	0.95	—	—	—	—
Methionine and cystine	2.50	3.00	0.43 ± 0.03	0.26 ± 0.02	0.36 ± 0.03	0.21 ± 0.01
Histidine	1.90	1.65	3.79 ± 0.05	2.17 ± 0.03	4.37 ± 0.05	2.50 ± 0.04
Arginine	—	2.10	—	—	2.00 ± 0.06	1.33 ± 0.03

^a^Suggested profile of essential amino acid requirements for adults (FAO/WHO, 1991) [27].

^b^Essential amino acid requirements of the common pig (3–10 kg) according to the NRC (1998) [28].

^c^Chemical score calculated using the FAO/WHO reference protein as the base.

^d^Chemical score calculated using the amino acid requirements as per the NRC (1998) [28].
